# Ischemic Myocardial Positron Emission Tomography Perfusion Without Epicardial Coronary Disease in Hypertrophic Obstructive Cardiomyopathy

**DOI:** 10.7759/cureus.52401

**Published:** 2024-01-16

**Authors:** Jason Galo, Rafey Feroze

**Affiliations:** 1 Cardiology, University Hospitals Cleveland Medical Center, Case Western Reserve University, Cleveland, USA

**Keywords:** refractory angina, microvascular angina, hypertrophic obstructive cardiomyopathy (hocm), pet scans, coronary vessel disease

## Abstract

The presence of impaired microvascular coronary flow (MCF) identified by positron emission tomography myocardial perfusion imaging (PET-MPI) has been described in hypertrophic obstructive cardiomyopathy (HOCM) patients, contributes to blunted myocardial perfusion during vasodilator stress, and is a strong predictor of poor prognosis. A 45-year-old female with hypertension and obesity presented with angina. Her PET-MPI displayed vasodilator stress-induced global LV ischemia. However, her coronary angiogram revealed no obstructive coronary disease. These contradictory findings triggered a more thorough cardiac MRI with diffuse myocardial fibrosis, indicating high-risk HOCM. She underwent implantable cardioverter-defibrillator (ICD) placement due to non-sustained ventricular tachycardia and syncope. While this patient lacked epicardial coronary disease, her PET-MPI demonstrated global LV ischemia due to decreased MCF, leading to inadequate augmentation of myocardial perfusion during hyperemia. This is a well-described phenomenon responsible for anginal symptoms in HOCM patients. HOCM hearts have abnormally thick coronary arterioles and decreased capillary density, leading to increased oxygen diffusion distances and reduced perfusion. The presence of vasodilator-induced, global ischemia on PET-MPI without epicardial stenosis should raise suspicion for HOCM with impaired MCF, which represents a high-risk population with an almost 10 times greater risk of cardiovascular mortality compared to hypertrophic cardiomyopathy with preserved MCF.

## Introduction

The presence of impaired microvascular coronary flow (MCF) identified by positron emission tomography myocardial perfusion imaging (PET-MPI) has been described in hypertrophic obstructive cardiomyopathy (HOCM) patients, contributes to blunted myocardial perfusion during vasodilator stress, and is a strong predictor of poor prognosis [1]. Here, we present a multimodality illustration of this phenomenon.

## Case presentation

**Figure 1:**
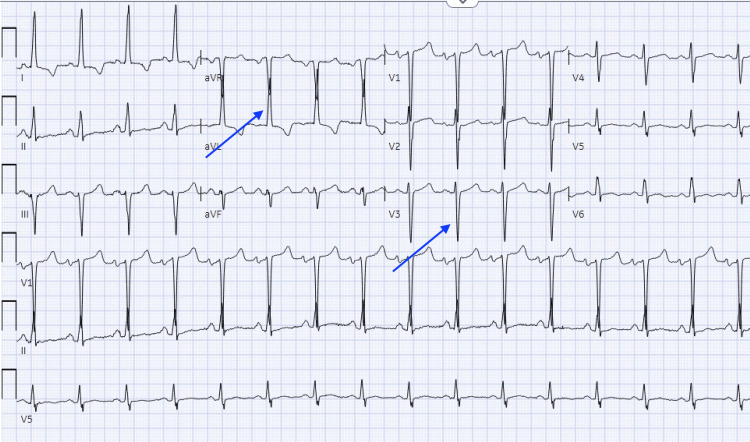
The EKG indicated left ventricular hypertrophy (LVH) according to the Cornell criteria, where the combined measurement of the R wave in aVL and the S wave in V3 surpassed 20 millimeters

A 45-year-old female with hypertension, obesity, active smoking, and obstructive sleep apnea (OSA) presented with exertional chest tightness and shortness of breath. At a height of 1.5 meters and weighing 152 kilograms, her BMI was calculated at 65.8, indicating severe obesity. Her blood pressure measured notably high at 205/97 mmHg, coupled with a heart rate of 76 beats per minute. Despite these concerns, her oxygen saturation remained at 98% while breathing room air. An electrocardiogram (ECG) showed normal sinus rhythm with left ventricular hypertrophy (LVH) and repolarization changes (Figure 1). 

**Figure 2:**
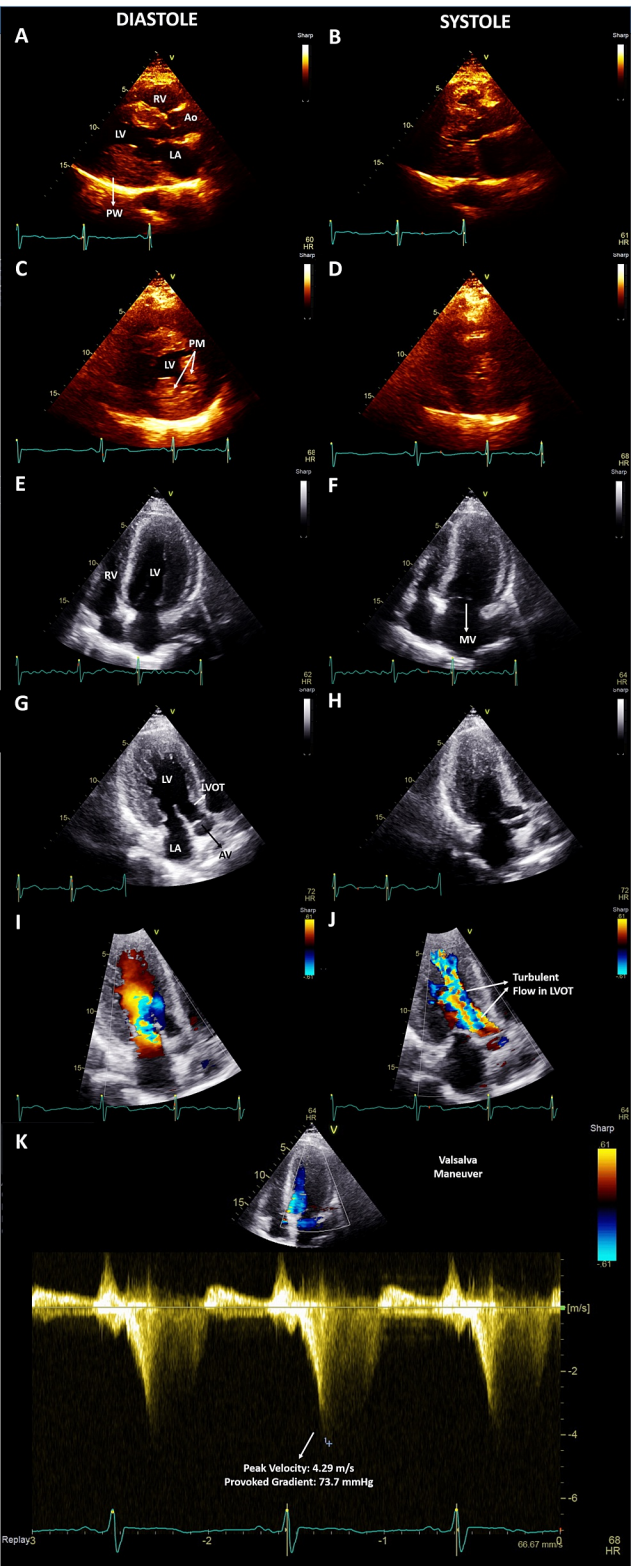
Transthoracic echocardiogram A transthoracic echocardiogram demonstrated severe left ventricular hypertrophy with thickened papillary muscles in the parasternal long (A, B), parasternal short (C, D), apical 4 chamber (E, F), and apical 3 chamber (G, H, I) views with preserved left ventricular ejection fraction and almost complete left ventricular cavity obliteration during systole. Impaired diastolic filling is present due to global left ventricular hypertrophy (not shown) along with abnormally turbulent flow in the left ventricular outflow tract during systole (J) due to septal hypertrophy without systolic anterior motion of the mitral valve. Continuous wave Doppler revealed resting and provoked gradients of 36 and 73 mmHg, respectively, across the left ventricular outflow tract (K) in the absence of aortic stenosis, confirming hypertrophic obstructive cardiomyopathy. RV - right ventricle; LV - left ventricle; Ao - aorta; LA - left atrium; PW - posterior wall; PM- papillary muscle; MV - mitral valve; AV - aortic valve; LVOT - left ventricular outflow tract

Troponin I was minimally elevated at 0.15 ng/L (the standard cutoff in our laboratory for normal levels is less than 0.04 ng/L), and brain natriuretic peptide (BNP) was elevated at 392 pg/mL. The echocardiogram revealed severe concentric LHV, showcasing an interventricular septal diameter measuring 2.2 cm, a left ventricular posterior wall thickness of 1.9 cm, and a relative wall thickness of 0.94. Additionally, the left ventricular mass index significantly elevated to 182 g/m2. Diastolic function was abnormal, characterized by a mildly dilated left atrium, trace tricuspid regurgitation (TR) impeding the estimation of tricuspid regurgitant velocity, an E/e' ratio of 22.30, and reduced medial and lateral e' velocities at 0.04 meters per second (Figure 2).

**Figure 3:**
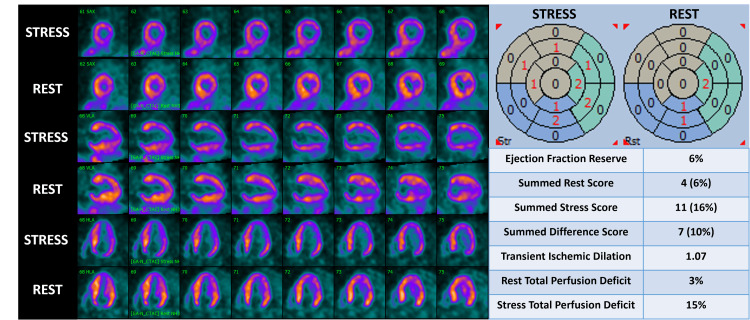
Positron emission tomography myocardial perfusion imaging (PET-MPI) using 13N-ammonia radiotracer exhibiting global ischemia with regadenoson-induced vasodilator stress, segment-based scoring showing a summed stress score of >10%, and increase in total perfusion deficit with stress

Given her low-risk ECG and non-concerning ischemic laboratory markers, she underwent a non-invasive ischemic assessment. The PET-MPI revealed vasodilator stress-induced global left ventricular ischemia (Figure 3).

**Figure 4:**
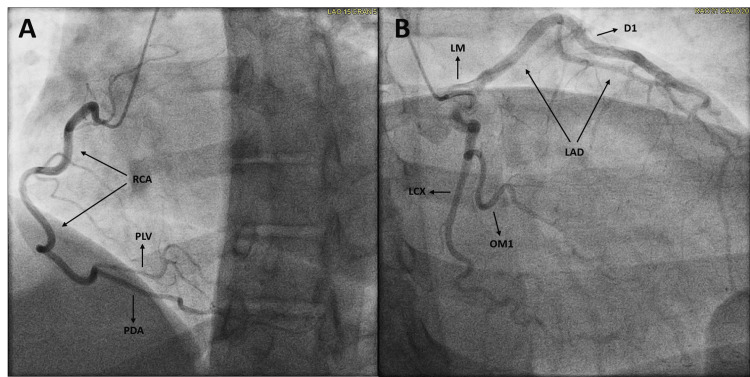
Coronary angiogram with an absence of obstructive coronary disease in the RCA (A), LM, LAD, LCX, (B) and their distal branches. Left ventricular end diastolic pressure of 24 mmHg was obtained RCA - right coronary artery; PLV - posterior left ventricular; PDA - posterior descending artery; LM - left main; LAD - left anterior descending; D1 - first diagonal; LCX - left circumflex; OM1 - first obtuse marginal

However, her coronary angiogram revealed no obstructive coronary disease (Figure 4). Although there are reports indicating that HOCM can lead to complications involving vasospasm, no specific provocative testing was conducted during the coronary angiogram procedure.

**Figure 5:**
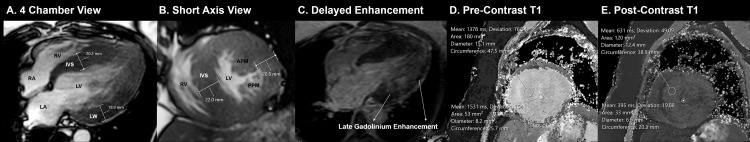
Cardiac magnetic resonance imaging Cardiac magnetic resonance steady-state precession imaging with diastolic parasternal long (A) and short axis (B) views showing severe, concentric, left ventricular hypertrophy with left ventricular ejection fraction of 63%, late gadolinium enhancement (C), and high extracellular volume of 31%, based on pre and post-contrast T1 values (D, E) and a hematocrit of 32%, altogether suggestive of myocardial fibrosis and high-risk hypertrophic cardiomyopathy. (IVS: interventricular septum, LA: left atrium, LV: left ventricle, LW: lateral wall, RA: right atrium, RV: right ventricle, APM: anterior papillary muscle, PPM: posterior papillary muscle)

These contradictory findings triggered a more thorough echocardiogram that demonstrated elevated left ventricular outflow tract (LVOT) gradients (Figure 1), confirming HOCM. Cardiac MRI showed diffuse myocardial fibrosis (scar size >13%), indicating high-risk HOCM (Figure 5).

The patient was initiated on carvedilol at 12.5mg twice daily, increased to 50mg twice daily, aiming for a heart rate below 70bpm. Despite this, syncopal episodes emerged. A four-week ECG monitor revealed three non-sustained ventricular tachycardia episodes. Considering age, 22mm wall thickness, 73mmHg LVOT gradient, syncope, and >13% late gadolinium enhancement (LGE) on MRI, she received an implantable cardioverter-defibrillator (ICD) (Class 2A indication). She experienced worsening exertional dyspnea, continued smoking, and medications for hypertension (Entresto, amlodipine, clonidine) were added. Refusal of mavacamten led to discussions on septal myectomy. A year and a half later, a repeat echocardiogram prompted referral for surgical myomectomy, but no surgery was performed due to transesophageal (TEE) findings of no systolic anterior motion of the mitral valve (SAM) or LVOT obstruction.

Subsequently, alcohol septal ablation resulted in improved resting and provoked gradients of 20mmHg and 32mmHg, respectively. Presently, she endures orthopnea and edema, hasn't had defibrillator shocks, and has started spironolactone and empagliflozin. Financial constraints hindered cardiac rehab. Further considerations involve mavacamten or repeat septal ablation if symptoms persist. 

## Discussion

When examining this case of hypertrophic obstructive cardiomyopathy (HOCM), the influence of accompanying cardiometabolic risk factors cannot be overlooked. The patient's profile, marked by obesity, obstructive sleep apnea, and a history of smoking, establishes a significant predisposition to microvascular coronary artery disease, irrespective of the HOCM diagnosis. These well-recognized risk factors play a pivotal role in the development of microvascular dysfunction, potentially exerting a considerable impact on the patient's cardiac condition.

Notably, despite the absence of epicardial coronary disease in this patient, the PET-MPI revealed global left ventricular (LV) ischemia, attributed to diminished myocardial perfusion augmentation during hyperemia, reflecting a recognized phenomenon responsible for anginal symptoms in HOCM patients with deranged coronary flow pathways [2-3]. It is imperative to acknowledge that these findings might not solely originate from HCM. Instead, the interaction between the hemodynamic properties of HOCM and the patient's cardiometabolic risk factors could exacerbate myocardial perfusion compromise.

Patients with HOCM often manifest diffuse microvascular dysfunction, prompting recurrent ischemic episodes despite the absence of coronary stenoses [4]. While the pathophysiological aspects of myocardial ischemia in HOCM have received attention, they remain relatively understated compared to the more prominently acknowledged mechanisms of LV outflow tract obstruction and diastolic dysfunction [5].

HOCM hearts typically exhibit abnormal thickening of coronary arterioles and reduced capillary density, leading to increased oxygen diffusion distances and diminished perfusion rates [6]. As left ventricular end-diastolic pressures rise, a concurrent decrease in coronary flow occurs [7]. Moreover, the patient's impaired ventricular relaxation and markedly elevated left ventricular end-diastolic pressure likely contributed to reduced myocardial perfusion pressure and subsequent ischemia [6]. The presence of vasodilator-induced global ischemia on PET-MPI, despite the absence of epicardial stenosis, should raise suspicion for HOCM with impaired myocardial coronary flow. This subgroup represents a high-risk population with a nearly tenfold higher cardiovascular mortality risk compared to hypertrophic cardiomyopathy cases with preserved myocardial coronary flow [1].

Moody et al. previously addressed a case akin to ours, delving into the intricate pathophysiological aspects contributing to small vessel ischemia [8]. Their comprehensive examination acknowledged several factors: the hypertrophy of intramural coronary arterioles, heightened regional wall stress linked to elevated intra-cavitary pressures causing flow impediment, disrupted cellular architecture marked by escalated collagen deposition in the interstitium, and ventricular hypertrophy correlating with reduced capillary density [8]. While our case mirrors similar pathophysiological elements, our study takes a more exhaustive approach in elucidating the nuanced mechanisms of microvascular dysfunction specific to hypertrophic obstructive cardiomyopathy (HOCM), as previously discussed.

## Conclusions

The presence of impaired microvascular coronary flow (MCF) identified by positron emission tomography myocardial perfusion imaging (PET-MPI) has been described in hypertrophic obstructive cardiomyopathy (HOCM) patients, contributes to blunted myocardial perfusion during vasodilator stress, and is a strong predictor of poor prognosis. HOCM hearts have abnormally thick coronary arterioles and decreased capillary density, leading to increased oxygen diffusion distances and reduced perfusion. The presence of vasodilator-induced, global ischemia on PET-MPI without epicardial stenosis should raise suspicion for HOCM with impaired MCF, which represents a high-risk population with an almost 10 times greater risk of cardiovascular mortality compared to hypertrophic cardiomyopathy with preserved MCF.

## References

[1] Cecchi F, Olivotto I, Gistri R, Lorenzoni R, Chiriatti G, Camici PG (2003). Coronary microvascular dysfunction and prognosis in hypertrophic cardiomyopathy. N Engl J Med.

[2] Ismail TF, Hsu LY, Greve AM (2014). Coronary microvascular ischemia in hypertrophic cardiomyopathy - a pixel-wise quantitative cardiovascular magnetic resonance perfusion study. J Cardiovasc Magn Reson.

[3] Raphael CE, Cooper R, Parker KH (2016). Mechanisms of myocardial ischemia in hypertrophic cardiomyopathy: insights from wave intensity analysis and magnetic resonance. J Am Coll Cardiol.

[4] Olivotto I, Cecchi F, Camici PG (2004). Coronary microvascular dysfunction and ischemia in hypertrophic cardiomyopathy. Mechanisms and clinical consequences. Ital Heart J.

[5] Maron MS, Olivotto I, Maron BJ, Prasad SK, Cecchi F, Udelson JE, Camici PG (2009). The case for myocardial ischemia in hypertrophic cardiomyopathy. J Am Coll Cardiol.

[6] Kofflard MJ, Michels M, Krams R, Kliffen M, Geleijnse ML, Ten Cate FJ, Serruys PW (2007). Coronary flow reserve in hypertrophic cardiomyopathy: relation with microvascular dysfunction and pathophysiological characteristics. Neth Heart J.

[7] Cannon RO 3rd, Rosing DR, Maron BJ, Leon MB, Bonow RO, Watson RM, Epstein SE (1985). Myocardial ischemia in patients with hypertrophic cardiomyopathy: contribution of inadequate vasodilator reserve and elevated left ventricular filling pressures. Circulation.

[8] Moody WE, Schmitt M, Arumugam P (2019). Coronary microvascular dysfunction in hypertrophic cardiomyopathy detected by Rubidium-82 positron emission tomography and cardiac magnetic resonance imaging. J Nucl Cardiol.

